# The Efficacy and Safety of Intravitreal Dexamethasone Implant for the Treatment of Macular Edema Related to Retinal Vein Occlusion: Real-life Data and Prognostic Factors in a Turkish Population

**DOI:** 10.4274/tjo.75317

**Published:** 2017-12-25

**Authors:** Ayşe Yağmur Kanra, Aylin Ardagil Akçakaya, Sevil Arı Yaylalı, Meltem Güzin Altınel, Neslihan Sevimli

**Affiliations:** 1 University of Health Sciences, Ümraniye Training and Research Hospital, Department of Ophthalmology, İstanbul, Turkey; 2 Medeniyet University, Göztepe Training and Research Hospital, Department of Ophthalmology, İstanbul, Turkey; 3 University of Health Sciences, Fatih Sultan Mehmet Training and Research Hospital, Department of Ophthalmology, İstanbul, Turkey; 4 Sultanbeyli State Hospital, Ophthalmology Clinic, İstanbul, Turkey

**Keywords:** Anti-vascular endothelial growth factor, intravitreal dexamethasone implant, macular edema, retinal vein occlusion

## Abstract

**Objectives:**

To evaluate the efficacy and safety of dexamethasone (DEX) implants as mono or combination therapy for macular edema in retinal vein occlusion (RVO) with real-life conditions, and to detect factors that influence final visual acuity.

**Materials and Methods:**

Twenty-five eyes with macular edema secondary to RVO underwent assessments for central macular thickness (CMT), best-corrected visual acuity (BCVA), adverse events, and also morphologic changes in optical coherence tomography at an interval of 4-8 weeks after at least one DEX implant.

**Results:**

Seventeen eyes with branch RVO and 8 eyes with central RVO were eligible for the study. The mean follow-up duration was 17 months (range, 12-26 months). Both mean BCVA (p=0.009) and CMT (p=0.006) improved significantly, and visual gains of ≥3 lines were achieved in 32% and ≥2 lines in 52% at the end of the follow-up period. The most powerful individual predictor of final visual acuity was baseline BCVA (r2=0.611, p<0.001, stepwise multiple regression), but the most efficient model was the combination of the ellipsoid zone (EZ) integrity and baseline BCVA (r2=0.766, p<0.001, stepwise multiple regression). Complication rates were very low after repeated DEX implants.

**Conclusion:**

DEX implant seems to be an effective and safe treatment for macular edema in RVO despite negative real-life factors, and visual outcomes are associated with baseline visual acuity and EZ integrity.

## INTRODUCTION

Retinal vein occlusion (RVO) is a common vascular disorder of the retina and the second most common cause of vision loss following diabetic retinopathy in industrialized countries.^1^ Macular edema is a common complication of both branch RVO (BRVO) and central RVO (CRVO) with or without ischemia.^2,3^

The pathogenesis of macular edema in RVO is not completely understood but previous studies have shown the role of hydrostatic effects from increased venous pressure and an increase in inflammatory cytokines such as interleukin-6 and prostaglandins, as well as vascular endothelial growth factor (VEGF). These lead to increased vascular permeability, vasodilatation, and breakdown of the inner blood-retina barrier due to dysregulation of endothelial tight junction proteins.^4,5,6^

The standard care for macular edema in BRVO was grid laser photocoagulation, and panretinal laser photocoagulation in the event of neovascularization; observation for macular edema was the only choice in CRVO. However, advances in retinal imaging and the pharmaceutical industry have radically changed the standard of care in the last decade.^4,5,6,7,8,9,10,11,12^

Steroids have potent anti-inflammatory effects; they inhibit the formation of both prostaglandins and leukotrienes, and decrease intracellular and extracellular edema by suppressing macrophage activity, reducing lymphokine production, downregulating the production of VEGF, and via their vasoconstrictive effect.13 After the SCORE study reported good short-term efficacy data on intravitreal triamcinolone acetonide both in terms of improving visual acuity and reducing central macular thickness (CMT) in patients with macular edema secondary to CRVO, observation was no longer an acceptable choice. Triamcinolone also had similar effectiveness when compared with grid laser for macular edema in BRVO.^8,9^ Ranibizumab, bevacizumab, and aflibercept as anti-VEGF agents, and steroids, especially dexamethasone (DEX) implants, are widely used in patients with RVO, marking a new epoch in the pharmacotherapy of macular edema via triamcinolone. Although bevacizumab remains an off-label treatment, DEX implant, ranibizumab, and recently aflibercept have all been approved.

The DEX implant contains 0.7 mg micronized preservative-free DEX in a biodegradable copolymer of polylactic-co-glycolic acid, which breaks down into carbon dioxide and water over time. It is designed to deliver drug to the retina over a period of up to 6 months. Intermittent release helps prevent peak vitreous drug concentrations and frequent repeat injections, thus the implant may potentially reduce the risk of unwanted steroid-related ocular adverse effects (cataract formation and intraocular pressure [IOP] elevation) and injection-related complications.^14^ A phase III clinical trial found DEX implant safe and effective in improving visual acuity and reducing the risk of vision loss when compared with a sham treatment.^10^ To assess the efficacy and safety of repeated DEX implants and to demonstrate factors that influence final visual acuity for macular edema in RVO, we selected a real-life setting for data collection.

## MATERIALS AND METHODS

Eighty-four eyes presenting with macular edema secondary to RVO and treated with DEX implants were reviewed in this interventional retrospective case series from one tertiary vitreoretinal care center between December 2013 and May 2016. The exclusion criteria were ischemic maculopathy, corticosteroid responders, epiretinal membrane visible on optical coherence tomography (OCT), naive eyes, history or presence of other maculopathies/retinopathies (e.g., age-related macular degeneration, uveitis), visually significant media opacities (e.g., cataract or corneal opacity), intravitreal anti-VEGF treatment within 1 month before DEX implant injections, and macular photocoagulation within 3 months before DEX implant injections. Therefore, the final evaluation included data from the remaining 25 eyes that met the study criteria. All eyes received DEX implants as a mono or combination therapy for the treatment of macular edema secondary to RVO with a minimum of 12 months follow-up and at least 3 months since the last DEX injection. Retreatment criteria were recurrence on OCT and loss of at least one line in BCVA. Retreatment was performed in accordance with Turkish National Health Insurance restrictions, which allows two DEX implants per year for this condition. Patients who did not meet this criterion were treated with an intravitreal ranibizumab injection and/or focal macular laser treatment until we were able to administer another DEX implant.

All patients included in the study underwent a complete ophthalmic examination: BCVA was assessed using the Early Treatment Diabetic Retinopathy Study chart at a distance of 4 m and then converted to logarithm of minimum angle of resolution (logMAR) units before statistical analysis. Demographic data, systemic diseases, treatments administered before DEX implant, anterior segment and fundus examination findings, and IOP measurements were collected from the patients’ files. The presence of macular and peripheral ischemia were evaluated at baseline and conversion of nonischemic to ischemic type and leakage for additional focal macular laser treatment were also evaluated using fluorescein angiography during follow-up. Peripheral retinal nonperfusion areas with evidence of neovascularization or high risk of its development (the presence of at least 10 disc areas of retinal capillary obliteration for CRVO and 5 disc areas for BRVO) underwent laser photocoagulation in ischemic RVO eyes. Macular OCT scans were performed using Topcon 3D OCT-2000 System; CMT measurements and featured macular morphology (subfoveal exudate plaques, the presence of serous macular detachment and RPE changes) were assessed at baseline and every 4-8 weeks after each injection by two retina specialists. The status of the ellipsoid zone (EZ) was also evaluated at the final visit as follows: (1) detected in the foveal area, intact; (2) detected as a disrupted line beneath the fovea; (3) lost in the fovea.^15^

Outcome measures included improvements in BCVA and CMT from baseline to last visit, the proportion of eyes with at least 3 lines of BCVA improvement, the proportion of eyes exhibiting ≥3 lines of BCVA worsening, and the incidence of adverse effects following repeated DEX implants. The presence and progression of lens opacities were assessed during slit-lamp examinations. Other local or systemic adverse events were also noted.

All patients underwent DEX implant injections in the operating room under subconjunctival anesthesia. They received topical moxifloxacin eye drops four times daily during the first week after injection and were examined on postoperative day 1 for visual acuity, anterior chamber reaction, IOP, and fundus evaluation using indirect ophthalmoscopy.

This study was approved by the local ethics committee and was conducted in accordance with the Declaration of Helsinki. Informed consent was obtained from all patients before injection.

### Statistical Analysis

Statistical analysis was performed using SPSS 20. The Shapiro-Wilk test was performed to test the normality of continuous variables. The paired t-test and Wilcoxon tests were used to compare the mean differences between pre- and post-implant values of all parameters evaluated (BCVA, CMT, IOP). The relative contribution of several variables, including SD-OCT characteristics such as the integrity of EZ and RPE changes at the final visit, presence of serous macular detachment at baseline, baseline BCVA, baseline CMT, and combined therapy applied were evaluated using stepwise multiple regression analysis. P values <0.05 were considered clinically significant results.

## RESULTS

Seventeen eyes with BRVO and 8 eyes with CRVO were eligible for the study. Ten (40%) patients were men. Most patients (68%) had hypertension, which is one of the most common risk factors for RVO. Chronic myeloid leukemia was diagnosed in one patient with CRVO when screening the etiology, and treatment with imatinib was started by the internal medicine department. Almost all patients had been treated previously for complications of RVO: 12 eyes had been treated with both anti-VEGF (ranibizumab or bevacizumab) and laser, 7 eyes only with anti-VEGF (ranibizumab or bevacizumab) injections, and 6 eyes only with laser treatment for macular edema. The baseline characteristics of the study population are summarized in Table 1.

The mean follow-up was 17.3±5 months (range, 12-26 months). A total of 64 DEX injections were administered during the study period (1 implant: 3 eyes, 2 implants: 11 eyes, 3 implants: 7 eyes, 4 implants: 2 eyes, and 5 implants: 2 eyes). The mean number of injections was 2.6±1.1. The mean recurrence time was 16.3±5.1 weeks (range, 12-28 weeks) for the first treatment, 13.5±2.8 weeks (range, 8-17 weeks) for the second treatment, and 13.5±2.6 weeks (range, 12-17 weeks) for the third treatment. Three eyes (12%) had no recurrence during follow-up with only one DEX implant. Peripheral photocoagulation for ischemia was performed in 3 eyes of the CRVO group and in 2 eyes of the BRVO group. Additional treatments included 10 eyes with both ranibizumab and focal macular laser, and 8 eyes only with ranibizumab injections. The mean number of ranibizumab injections was 1.8±1.5 (maximum 5). Seven eyes were treated with DEX implant monotherapy.

Both mean BCVA (p=0.009) and CMT (p=0.006) improved significantly at the final visit. The preoperative mean CMT was 539±165 µm, which decreased to 246±118 µm. In accordance with the OCT changes, the preoperative mean BCVA improved from 0.72±0.27 (logMAR) to 0.59±0.32 (Table 2). From the first to the fourth injection, BCVA improvement of at least 3 lines within 3 months was seen in 52%, 36%, 27%, and 33% of the eyes, respectively. The proportion of eyes demonstrating ≥3 lines visual gain was 32% and ≥2 lines gain was 52% at the end of the follow-up period. No eyes showed ≥3 lines of worsening. Two eyes showed BCVA reduction of nearly 1 line compared to baseline.

**Figure 1:**
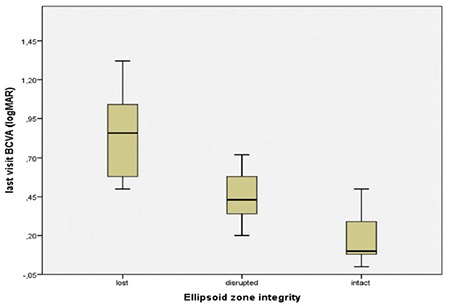
The relationship between ellipsoid zone integrity and visual outcomes; final best-corrected visual acuity was worse in eyes with lost integrity than in the other two groups BCVA: Best-corrected visual acuity, LogMAR: Logarithm of minimum angle of resolution

The most powerful individual predictor of final BCVA among patients with macular edema secondary to RVO was baseline BCVA (r2=0.611, p<0.001, stepwise multiple regression). However, the most efficient model was the combination of EZ integrity and baseline BCVA (r2=0.766, p<0.001, stepwise multiple regression). The EZ was intact in only 7 eyes, disrupted in 10 eyes, and lost in 8 eyes due to prolonged edema (Figure 1). This was not associated with CMT values at baseline or at the final visit (p=0.20); no other factors were associated with final BCVA.

**Figure 2:**
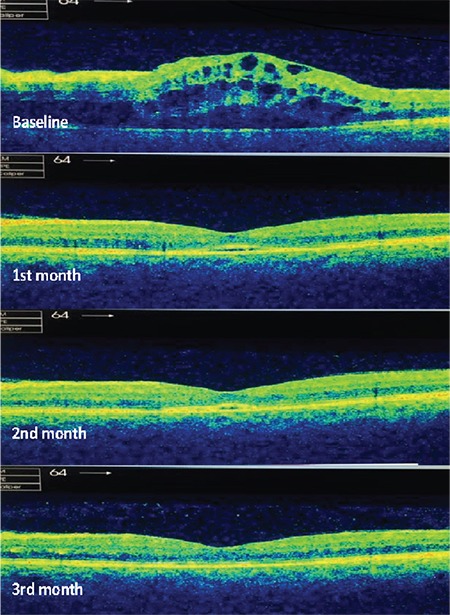
Representative optical coherence tomography images of complete regression of submacular detachment with macular edema after a single dexamethasone implant in a patient with central retinal vein occlusion within three months

**Figure 3:**
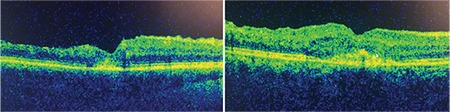
Presence of retinal pigment epithelium changes are seen in the fovea as a result of chronicity after regression of the macular edema following dexamethasone implants in the two different patients

There was submacular detachment (SMD) in 11 eyes at baseline (Figure 2). SMD generally tended to have lower height and existed for a shorter duration when developing in cases of recurrence in these 7 eyes. There were extensive subfoveal exudate plaques in 3 eyes at baseline, which regressed accompanying improvements in BCVA during follow-up with repeated DEX implants. There were subfoveal RPE changes (atrophy or hypertrophy) on OCT accompanying disrupted or lost EZ in 6 eyes (Figure 3). During follow-up, newly developed retinal vein occlusions were found in the fellow eyes of two of the study patients.

A rebound effect, characterized by a late increase in CMT to an excess of the baseline level, occurred in 4 eyes at months 3 and 4. We only evaluated the rebound effect for DEX implants and not for combined therapies. The rebound phenomenon was not a negative factor in functional or anatomic recovery when retreatment was provided.

No serious ocular or systemic adverse events were observed after repeated DEX implants. We observed a fragmented DEX implant in one BRVO eye, but fragmentation did not cause clinically significant effects. The IOP values of all patients were within normal range (<21 mmHg) at the initial visit. During the study period, 36% of eyes exhibited IOP higher than 25 mmHg (maximum 32 mmHg) and 32% showed an increase in IOP of at least 10 mmHg over baseline at 1 or more visits. All cases were treated and well controlled with a maximum of three IOP-lowering agents. No additional treatment (laser or surgery) was required. IOP rises were usually transient except in two (8%) patients, one of whom had PEX syndrome while the other had a family history of glaucoma. IOP was kept under control only with implantation of an Ahmed Glaucoma valve and intravitreal ranibizumab injections in a patient with ischemic CRVO due to neovascular glaucoma. Significant cataract progression was observed in 8 (32%) eyes after second or third implants; cataracts were extracted at the investigator’s and patient’s discretion in a total of 7 study eyes. There were no injection-related complications such as endophthalmitis or retinal tears or detachment.

## DISCUSSION

Randomized controlled trials support the fact that anti-VEGF agents and DEX implants may be used as a first-line therapy for macular edema secondary to RVO.^10,11,12^ In addition, laser photocoagulation can contribute by reducing the number of intravitreal injections in appropriate cases. For example, Pichi et al.16 investigated monotherapy versus combination therapy with macular grid laser in 50 patients with BRVO. The combination group was better than the monotherapy group in visual acuity outcomes (0.32±0.29 logMAR, 0.18±0.14 logMAR) and had longer intervals between injections with fewer implants.

We have to perform combination therapies in most difficult-to-treat patients because the Turkish social security system limits DEX implants to two per year and anti-VEGF agents to seven over the lifetime of each patient. It was reported that obtaining clinically significant anatomic and functional outcomes was harder in patients with longer duration and repeated treatments compared to naive eyes.^15,17^ In this retrospective case series, preoperative mean BCVA significantly improved from 0.72 to 0.59 logMAR with a concomitant decrease in retinal thickness similar to previous reports at the final visit.^10,17,18,19^ The favorable effect of repeated DEX implants on both was consistent and showed durability over repeat injections (Table 2). The proportion of eyes demonstrating ≥3 lines gain was 32% and ≥2 lines gain was 52% at the end of the follow-up period, consistent with the Shasta study (including 285 patients treated with multiple DEX implants for macular edema secondary to RVO); 34% of eyes achieved at least 3 lines of improvement in BCVA and 46% achieved at least 2 lines from baseline after each of the first 6 implant injections. Our study found that no eyes showed ≥3 lines of decline but two eyes showed a decline in BCVA of nearly 1 line compared with baseline. Although decreases in CMT values were obtained after repeated DEX implants in these patients, the lost integrity of EZ and foveal atrophy affected the final visual acuity unfavorably.

In the GENEVA study, injections were not performed before 6 months, and the treatment interval was not clear because the study prioritized the safety and efficacy evaluation of 1 or 2 treatments with DEX implants over 12 months in eyes with macular edema secondary to RVO. In real-life clinical studies, Coscas et al.^17^ found the mean interval for DEX injection as 5.9 months following the first injection and 8.7 months for the second, whereas it was 5.6 months in the Shasta study.^18^ Joshi et al. ^19^ observed the time to retreatment as 17 weeks in BRVO, 18 weeks in CRVO, and with repeated injections it decreased to 10 weeks. We could only evaluate the recurrence interval after each DEX injection, not the reinjection intervals due to the combined therapeutic approach used with our patients. Consistent with the aforementioned study, we observed that the interval shortened, with recurrence occurring 16 weeks after the first implant and 13.5 weeks after the second and third. We think that this course was not associated with tachyphylaxis but might be related with starting therapy with a more aggressive disease and insurance issues because we could not perform regular DEX implants.

We evaluated the relationship between final BCVA and EZ status and RPE changes at the final visit, presence of serous macular detachment at baseline, baseline BCVA, baseline CMT, and a combined therapy approach. We observed that final visual outcomes were associated with both baseline BCVA and EZ status. It is widely recognized that EZ integrity, which is an important indicator of photoreceptor function, has a close relationship with better final visual acuity.^20,21^ The presence of intact EZ in only 28% of eyes in the present study is attributable to prolonged macular edema and irreversible tissue damage.

The mechanism of developing SMD is unclear but is thought to be different from diabetic macular edema. It is claimed to be associated with hydrostatic pressure increase within retinal vessels, which results in drainage failure. This causes strain on Müller cells, and the resulting inner traction forces lead to detachment.^22^ Moreover, different rates of SMD have been reported in previous studies, probably based on the resolution of OCT devices. In the current study, we used a Topcon 3D OCT-2000 System and the SMD rate was high (44%) at baseline. Maggio et al.15 observed that SMD was a negative prognostic factor, although it did not prevent the regression of macular edema. The presence of SMD at baseline was not prognostic for final BCVA in our series. Additionally, recurrent SMD after DEX injections tended to have lower height and shorter duration.

Chronicity of edema may lead to RPE changes overlooked on fundus examination but can be clearly revealed as hyperreflective foci underneath the fovea on OCT. They were mostly accompanied by EZ defects and were argued to be a prognostic factor, like EZ, in the long-term follow-up of patients who were treated with ranibizumab or DEX implants for RVO.^23,24^ Farinha et al.^23^ found that baseline BCVA and disruption of the RPE were predictors of final BCVA. Additional investigations on larger numbers of eyes are needed to better understand the prognostic effects of SMD and RPE changes for macular edema in RVO.

Common complications of ocular corticosteroid therapy are IOP elevation and cataract formation/progression. DEX is less lipophilic than fluocinolone acetonide and shows less sequestration in the lens and trabecular meshwork, and so it is thought that DEX implant has potentially lower risk of causing IOP elevation and cataract.^25^ IOP increases were moderate in severity, easily managed with IOP-lowering medication, and generally transient. No additional treatment with laser or surgery was required in our patients.

In the GENEVA study, 29.8% cataract progression was observed in patients who received two 0.7 mg DEX implant injections versus 5.7% in the sham-treated phakic eyes over 12 months. Cataract surgery was performed in 1.3% of the DEX-treated and 1.1% of the sham-treated eyes.^10^ However, in the MEAD study, there was a 60% rate of crystalline lens surgery at 3 years, and the authors claimed that cataract surgery could have been underestimated in the GENEVA trial.^26^ The timing of cataract surgery may have been postponed in most studies in order to exclude Irvine-Gass syndrome or other possible causes that might affect the results of macular edema and the course of the study. Gradual cataract progression was observed after repeated implants and cataracts were extracted at the investigator’s and patient’s discretion in 28% of the eyes in the present study. This somewhat high rate of cataract may lead to concerns in patients with phakic eyes. However, we assume that it should not be a barrier to repeated DEX implant use in patients with RVO because modern cataract extraction is a safe procedure.

### Study Limitations

Our study has several limitations, including its retrospective nature and small study population without separation of BRVO and CRVO results. Moreover, insurance issues prevented us from administering DEX implants whenever it was indicated, which forced us to choose different treatment strategies. However, we think that this study presents valuable real-life clinical data in a Turkish cohort. Preservation and even gain of vision were achieved in most individuals, and prognostic factors affecting final visual outcomes and morphologic findings on OCT were also evaluated.

## CONCLUSION

Ellipsoid zone integrity on OCT and basal visual acuity might give clues for visual outcomes in DEX implant treatment of macular edema secondary to RVO. Combination therapies can provide functional and anatomic results equivalent to those achieved in DEX monotherapy in real-life clinical settings.

## Figures and Tables

**Table 1:**
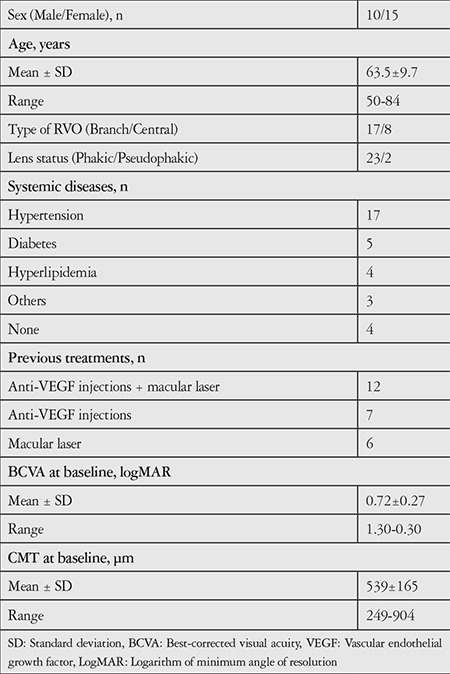
Baseline characteristics of the study population

**Table 2:**
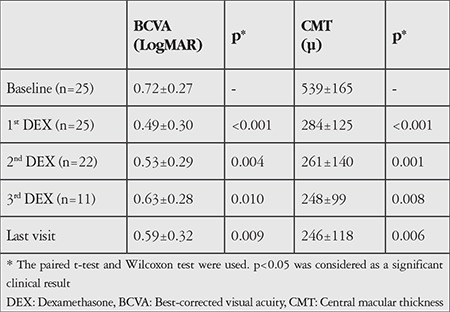
The mean changes in best-corrected visual acuity and central macular thickness values at month 2 after each dexamethasone implant as a mono or combination therapy compared with baseline
